# Drivers’ behavior confronting fixed and point-to-point speed enforcement camera: agent-based simulation and translation to crash relative risk change

**DOI:** 10.1038/s41598-024-52265-3

**Published:** 2024-01-22

**Authors:** Seyed Amir Ahmad Safavi-Naini, Shayan Sobhani, Mohammad-Reza Malekpour, Kavi Bhalla, Saeid Shahraz, Rosa Haghshenas, Seyyed-Hadi Ghamari, Mohsen Abbasi-Kangevari, Nazila Rezaei, Seyed Taghi Heydari, Negar Rezaei, Kamran B. Lankarani, Farshad Farzadfar

**Affiliations:** 1 Non-Communicable Diseases Research Center, Endocrinology and Metabolism Population Sciences Institute, Tehran University of Medical Sciences, Tehran, Iran; 2 Research Institute for Gastroenterology and Liver Diseases, Shahid Beheshti University of Medical Sciences, Tehran, Iran; 3 Independent Researcher, Waterloo, Ontario Canada; 4 Public Health Sciences, University of Chicago, Chicago, IL USA; 5 Institute for Clinical Research and Health Policy Studies, Tufts Medical Center, Boston, MA USA; 6 Health Policy Research Center, Institute of Health, Shiraz University of Medical Sciences, Shiraz, Iran; 7 Endocrinology and Metabolism Research Center, Endocrinology and Metabolism Clinical Sciences Institute, Tehran University of Medical Sciences, Tehran, Iran

**Keywords:** Trauma, Epidemiology, Statistics, Disease prevention, Health policy, Public health, Epidemiology

## Abstract

Utilizing a novel microsimulation approach, this study evaluates the impact of fixed and average point-to-point Speed Enforcement Cameras (SEC) on driving safety. Using the SUMO software, agent-based models for a 6-km highway without exits or obstacles were created. Telematics data from 93,160 trips were used to determine the desired free-flow speed. A total of 13,860 scenarios were simulated with 30 random seeds. The ratio of unsafe driving (RUD) is the spatial division of the total distance travelled at an unsafe speed by the total travel distance. The study compared different SEC implementations under different road traffic and community behaviours using the Power Model and calculated crash risk changes. Results showed that adding one or two fixed SECs reduced RUD by 0.20% (0.18–0.23) and 0.57% (0.54–0.59), respectively. However, average SECs significantly lowered RUD by 10.97% (10.95–10.99). Furthermore, a 1% increase in telematics enforcement decreased RUD by 0.22% (0.21–0.22). Point-to-point cameras effectively reduced crash risk in all implementation scenarios, with reductions ranging from − 3.44 to − 11.27%, pointing to their superiority as speed enforcement across various scenarios. Our cost-conscious and replicable approach can provide interim assessments of SEC effectiveness, even in low-income countries.

## Introduction

Transportation injury is the leading cause of death among children and young adults, accounting for one in every six fatalities within this demographic. As of 2019, road traffic injuries (RTIs) contributed to 4.2% of global disability-adjusted life years (DALYs), marking a 3% increase since 1990^1^. The World Health Organization (WHO) declared the need for strengthening legislation, especially in low- and middle-income countries where 93% of fatality occurs^2,3^. RTIs pose an even greater concern in developing countries, owing to the rapid expansion of urban areas and the uneven development of infrastructure^3^. In Iran and other Middle Eastern countries, road accident fatalities are twice the global average, underscoring the urgent need for preventive measures in the public health sector^1^.

Preventing RTIs requires a multifaceted approach, including law enforcement, vehicle safety, post-accident care, and education^3,4^. Speed is a key risk factor in crash mortality, significantly affecting the likelihood and severity of RTIs^5^. Consequently, preventive interventions mainly focus on reducing vehicle speed at the time of a crash. Among these strategies, speed enforcement cameras (SECs) are widely used^5^. The two most common types are fixed SECs and average SECs (also known as point-to-point speed cameras). Recent studies has raised questions about the effectiveness of fixed SECs, noting that over 50% of drivers continue to exceed speed limits even after installation of SECs^6^.

New telematic-based enforcements methods offer potential for improved strategies. In these systems, data from a vehicle-installed Global Positioning System (GPS) are transmitted to a central system. This digital blueprint allows for tracking vehicle speed and applying necessary incentives^7^. These systems serve as enforcement tools by analyzing data, detecting errors, and predicting crashes, influencing driver behavior through feedback and incentives^8–11^. Incentives, such as altered insurance fees or driver charges, have proven effective in reducing crash incidents by 38%^11,12^.

Beyond enforcement, in-vehicle devices contribute to investigating driving behavior, as exemplified by previous studies like the Strategic Highway Research Program 2 (SHRP2-NDS) utilizing GPS-based data, video records, and sensor data^9,13^. Despite providing rigorous data, evidence and best practices for cost-effective enforcement tools remain limited, particularly in resource-constrained developing countries.

The WHO noted a lack of robust evidence for interventions targeting RTI prevention in 2004. Still, many unanswered questions exist in the literature^4,14,15^. Existing studies often rely on before-after comparisons, raising validity concerns and potential biases in interrupted time series analysis^5,15^. Additionally, the effectiveness of speed cameras may diminish over time as drivers learn to circumvent them (time halo effect), and the phenomenon of slowing down before and accelerating after cameras, known as the distance halo effect or kangaroo driving, has been observed^15,16^. This V shape declaration before and acceleration after the camera is called the distance halo effect or kangaroo driving^17^. Despite ongoing debates, more accurate methods are needed to generate evidence for policymaking^15^.

Addressing the research-practice gap in road safety interventions requires innovative methods^18^. Developing real-world methodologies to accurately assess driver behaviors can be a laborious, costly, and occasionally impractical undertaking, as indicated by significant participant dropout rates observed in our studies and others^19^. This challenge is further exacerbated by the inclination of reckless drivers to discontinue their participation. Simplified designs, such as before-after studies, may lack robust validity^5^. Simulations offer a controlled environment to predict behavior and, if conducted properly, can yield valid results. Agent-based models emerge as potent tools for simulating individual behavior, with extensive applications in diverse fields, including sociology, human traffic, and driver behavior^20–26^. This study aims to assess the effectiveness of fixed SECs, average SECs, and telematic installations in promoting safe driving through agent-based modeling. Subsequently, our goal is to translate simulation results to compare changes in crash risk under various scenarios, thereby enabling the implementation of cost-effective enforcement strategies tailored to the specific context of deployment.

## Material and methods

### Data collection (real-world phase)

This study utilized data sourced from telematics devices installed in taxis operating at the three busiest terminals in Fars province, Iran, over a five-month period starting in September 2021. All potential participants were provided with a flier that described the purpose of the study, and drivers who gave written consent were included in the study. The telematics devices used in this study recorded the GPS coordinates of the vehicles, along with their speed and 3-axis acceleration every 10 s. Subsequently, this data was transmitted to the data center via the built-in Global System for Mobile Communications (GSM) module within the devices.

### Driver categories

Drivers were categorized based on their worst behavior in proximity to SECs. The 95th percentile of recorded speeds on highways with a 90 km/h speed limit was defined as the Desired Free-Flow Speed (DFFS) for each driver, representing their chosen speed under low traffic conditions without speed-reducing factors. This data was used to calculate the DFFS distribution for each driver group^17,27^.

Driver categories included: (A) Law-abiding drivers, (B) Kangaroo drivers, and (C) Consistent-speeders, as defined by prior studies^17,28,29^. In particular categories of drivers consists of (1) law-abiding cautious drivers who obey speed limits without requiring any additional controls or incentives (Group A: law-abiding), ^2^ drivers who drive over the speed limit but decrease their speed abruptly in the zone of speed enforcement cameras to avoid potential penalties (Group B: kangaroo driver), and (3) drivers who drive over the speed limit regardless of the existence of speed control cameras and potential penalties (Group C: consistent speeders).

### Agent-based modelling and scenarios

In this simulation study, we evaluated the sensitivity of drivers' behavior to the combined effect of telematics devices and SECs by modeling various scenarios. To achieve this objective, we utilized Simulation of Urban Mobility (SUMO), an open-source microsimulation software package, to develop the necessary Agent-Based models^30^. We implemented a 6-km highway segment in SUMO with no exit, entry, turn, or driving obstacles to construct the model. sed on the simulation scenario, four types of SECs were deployed:

1. One fixed SEC at the middle of the segment;
2. Two fixed SECs at the first and second thirds of the segment;
3. Three fixed SECs at the first, second, and third quartiles of the segment;
4. Average (point-to-point) SECs at the first and third quartiles of the segment.

**Figure 1:**
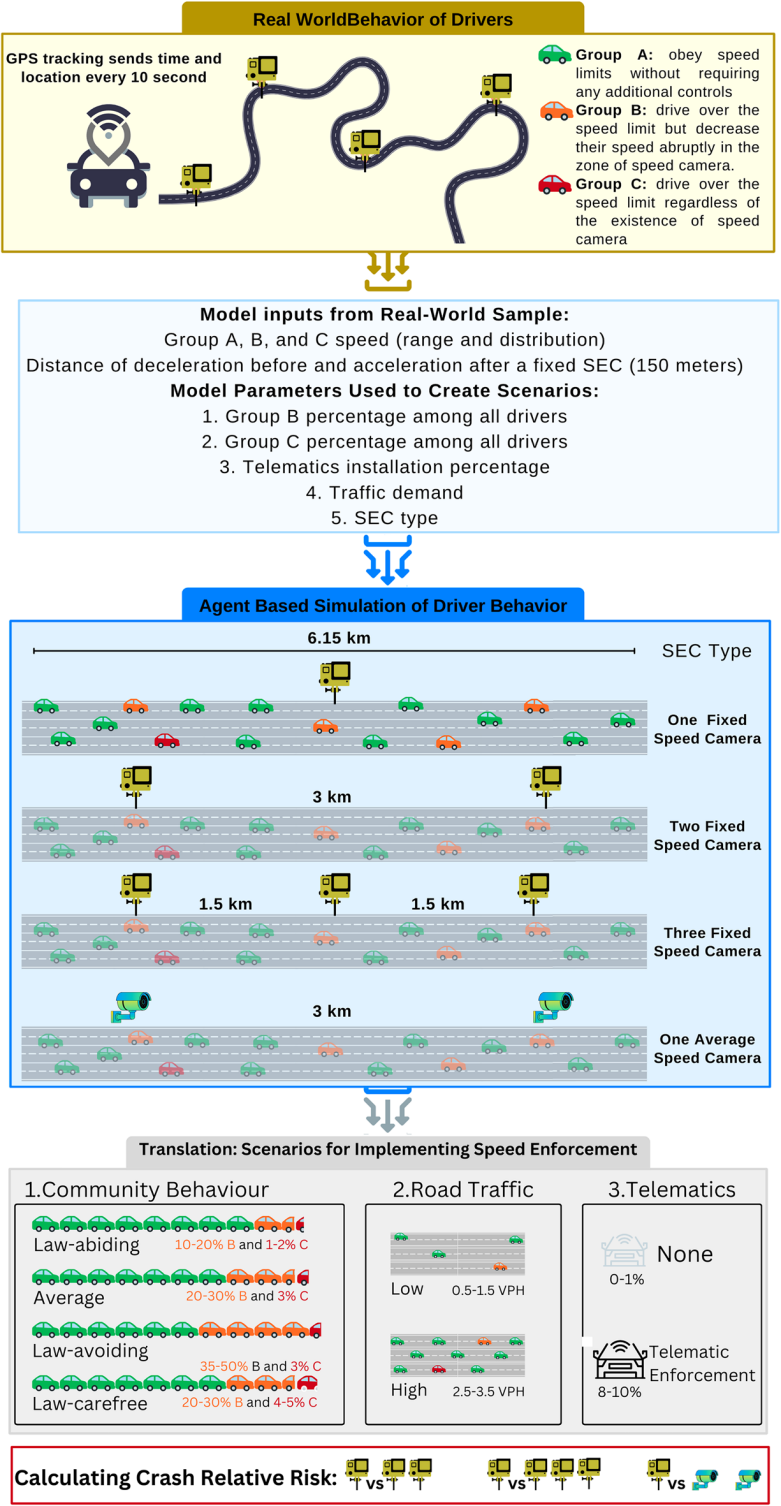
Study population, agent-based modelling parameters, and method of translating simulation results into practice by defining implementation scenarios. (Footnote: GPS, Global Positioning System; VPH, vehicle per hour).

The detection range for fixed SECs was 150 m before the cameras. Figure 1 provides a brief overview of the method and implementation of our agent-based models.

### Simulation variables

Scenarios were formulated based on five key variables: type of SEC, percentage of telematics installation for Groups B and C, proportion of Group B drivers, proportion of Group C drivers, and traffic demand, as illustrated in Fig. 1. Telematics devices were randomly installed in 0% to 10% of vehicles driven by Group B and C drivers to enforce speed limit adherence. This setup aimed to replicate a real-world situation where telematics is used as an enforcement tool for speed limit violators. The representation of Group B was varied from 10 to 50% in increments of 5%, and Group C from 1 to 5% in increments of 1%. Traffic demand was defined in terms of vehicles per hour (VPH), ranging from 500 to 3500 VPH in 500 VPH increments. A total of 13,860 scenarios were simulated using 30 random seeds, resulting in 415,800 individual simulations.

Spill-over in traffic simulation refers to the effect where changes in traffic flow or behavior in one area extend to and impact adjacent areas or lanes This can occur when alterations in traffic conditions, such as increased congestion, affect traffic patterns. To investigate the possibility of a spill-over effect of telematics installation, we also simulated high percentages of telematics installation, ranging from 10 to 90%, for traffic demands of more than 1500 VPH and the average percentage of groups B and C (30% and 3%, respectively).

### Translating simulation result to crash risk

We translated our results, which comprised 415,800 simulations, into changes in crash risk by calculating the mean speed change and relating it to crash risk change using the Power Model. The Power Model is a mathematical formula employed to estimate the relationship between vehicle speed and the likelihood or severity of a crash^5^. It proposes that the risk of a crash, or its severity, increases exponentially with speed with great precision^31^. The model is typically represented as:

$$Crash \;Risk \propto Speed^{n}$$

We assumed the n of 3, representing the relationship between speeding with fatal and serious injuries, to compare crash relative risk between two scenarios^31^:

$$Crash \;Relative\; Risk = \left( {\frac{Mean\; Speed\; in \;Scenrio \;2}{{Mean \;Speed \;in \;Scenrio \;1}}} \right)^{3}$$

Under different scenarios for implementing SECs, four types of SEC interventions were compared using 1 fixed camera as the reference (i.e., 2-camera vs. 1-camera; 3-camera vs. 1-camera; point-to-point camera vs. 1-camera). In particular, for each variable, groups were defined if a step size in mean speed was observed during the exploratory phase. Using this approach, the variables that resulted in different scenarios were:

1. Traffic (demand):
  - Low (0.5–1.5 VPH)
  - Moderate (2.5–3.5 VPH)
2. Telematic installation:
  - None (0–1%)
  - Telematic enforcement for law violators (8–10%)
3. Community Behavior:
  - Law-abiding community which abides by the law due to internal factors (i.e., culture) or external factors (i.e., effective enforcement): 10–20% Group B and 1–2% Group C
  - Average community consisting of some extent of law-tricksters (Group B) and some extent of law-offenders (Group C), similar to our real-world context in Iran: 20–30% Group B and 3% Group C
  - Law-avoiding community consisting of a high number of tricksters: 35–50% Group B and 3% Group C
  - Law-carefree community consisting of a high number of law offenders due to ineffective enforcements: 20–30% Group B and 4–5% Group C

### Statistical analysis

In this study, a speed that exceeds 90 km/h was defined as unsafe driving speed. The computation of the ratio of unsafe driving (RUD) as the division of total distance traveled at unsafe speed by total traveled distance helped compare different scenarios. The linear regression analysis with an alpha of 0.05 was utilized to infer the statistical significance of the variables' association with RUD. The change in crash relative risk was calculated in percentage after estimating crash relative risk using the previously mentioned formula. All statistical analyses were performed using Statsmodels, version 0.13, an open-source Python library^32^. Apache Spark, version 3.0, a unified big data processing engine, was utilized for data preprocessing^33^.

### Ethics statement

The study was conducted according to the guidelines of the Declaration of Helsinki and approved by the Research Ethics Committees of the National Institute for Medical Research Development (IR.NIMAD.REC.1399.032, 2019-12-07). All participants signed a written informed consent form allowing their driving data to be used anonymously and confidentially in research projects.

## Results

**Figure 2:**
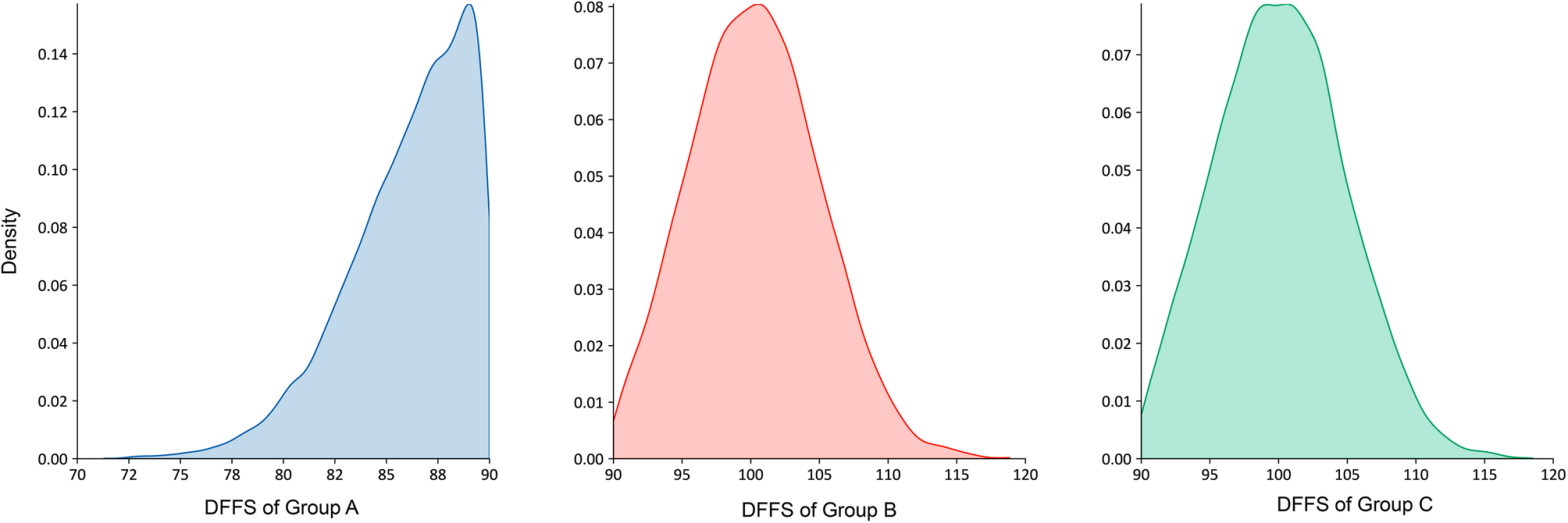
Desired Free-Flow Speed (DFFS) among three identified groups of taxi drivers' behavior.

A total of 397 male taxi drivers, with a mean age of 46.88 (standard deviation: 10.12), consented to participate in the study. Driving behavior of 93,160 trips were used as an input of simulations. Among participants, 92 (23.17%) were categorized as Group B, and 13 (3.27%) were categorized as Group C. The distribution of DFFS for each group of drivers is shown in Fig. 2**.**

### SECs impact on RUD

The fitted linear regression with all the variables of the simulation, resulted in an adjusted R-squared value of 0.925 (Table 1). Compared to one fixed SEC, the installation of one and two additional fixed SECs reduced RUD slightly by 0.20% (0.18–0.23) and 0.57% (0.54–0.59), respectively. In contrast, installing average SECs resulted in a significant decrease of 10.97% (10.95–10.99) in RUD.

**Table 1:** Variables and results of the linear regression analysis (SEC, speed enforcement camera; VPH, vehicle per hour.

	Coefficient (95% confidence interval)	Standard error	t-value	p-value
Two fixed SECs	− 0.2 (− 0.23 to − 0.18)	0.01	− 16.78	< 0.001
Three fixed SECs	− 0.57 (− 0.59 to − 0.54)	0.01	− 47.07	< 0.001
Average SECs	− 10.97 (− 10.99 to − 10.95)	0.01	− 907.32	< 0.001
Vehicle count (1000 VPH)	− 3.97 (− 3.98 to − 3.96)	< 0.01	− 929.41	< 0.001
Group B drivers (Percent)	0.61 (0.61 to 0.61)	< 0.01	1830.57	< 0.001
Group C drivers (Percent)	0.68 (0.68 to 0.69)	< 0.01	225.74	< 0.001
Telematics installation (Percent)	− 0.22 (− 0.22 to − 0.21)	< 0.01	− 161.01	< 0.001

### Driver categories: A, abiding-law; B, kangaroo driver; C, consistent-speeder

Each 1% increase in the number of groups B and C resulted in 0.61% (0.61–0.61) and 0.68% (0.68–0.69) more RUD, respectively. The mean RUD was 0.56 (0.56–0.56) for group B and 0.65 (0.65–0.65) for group C. Although average SEC significantly reduced the RUD in Group B compared to Group C (29.34% vs. 58.36%), the impact of fixed SECs was neglectable, with RUD values at 64.86% for Group B and 67.21% for Group C.

### Telematic installation impact on RUD

Overall, each 1% increase in telematics installation resulted in a 0.22% (95% Confidence Interval: 0.21–0.22%) decrease in RUD. Besides, in the setting of average SECs, the association of telematics installation with a reduction in RUD was less pronounced compared to fixed SECs (0.12% vs. 0.25%).

### Traffic impact on RUD

**Figure 3:**
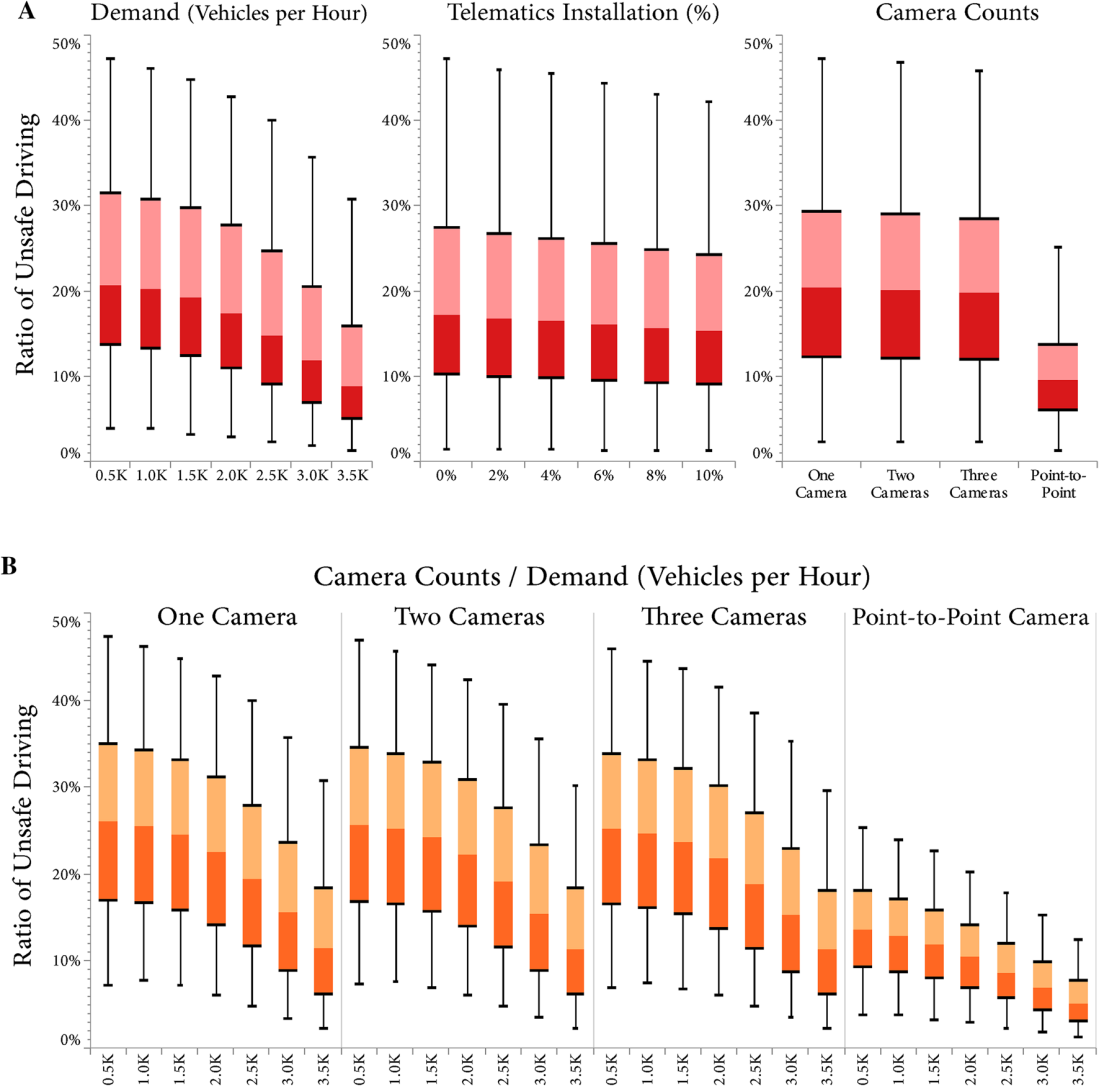
Ratio of unsafe driving in different settings of vehicle demand, telematic installation, camera count, and camera type.

**Figure 4:**
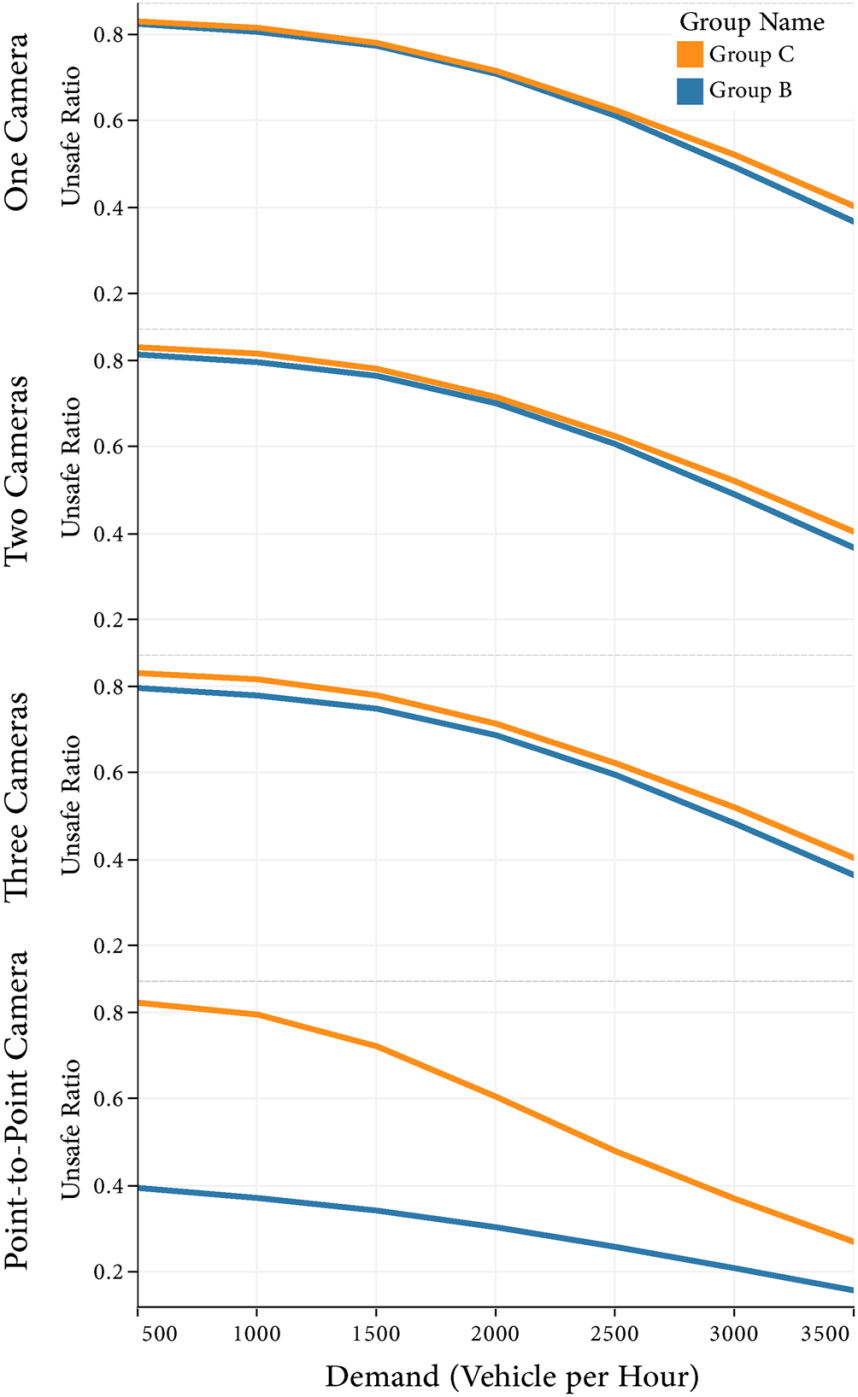
Unsafe driving ratio accounting for vehicle demands in different combination of speed cameras (Group B: drive over the speed limit but decrease their speed abruptly in the zone of camera (Kangaroo driving); Group C: exceed the speed limit regardless of enforcements).

**Figure 5:**
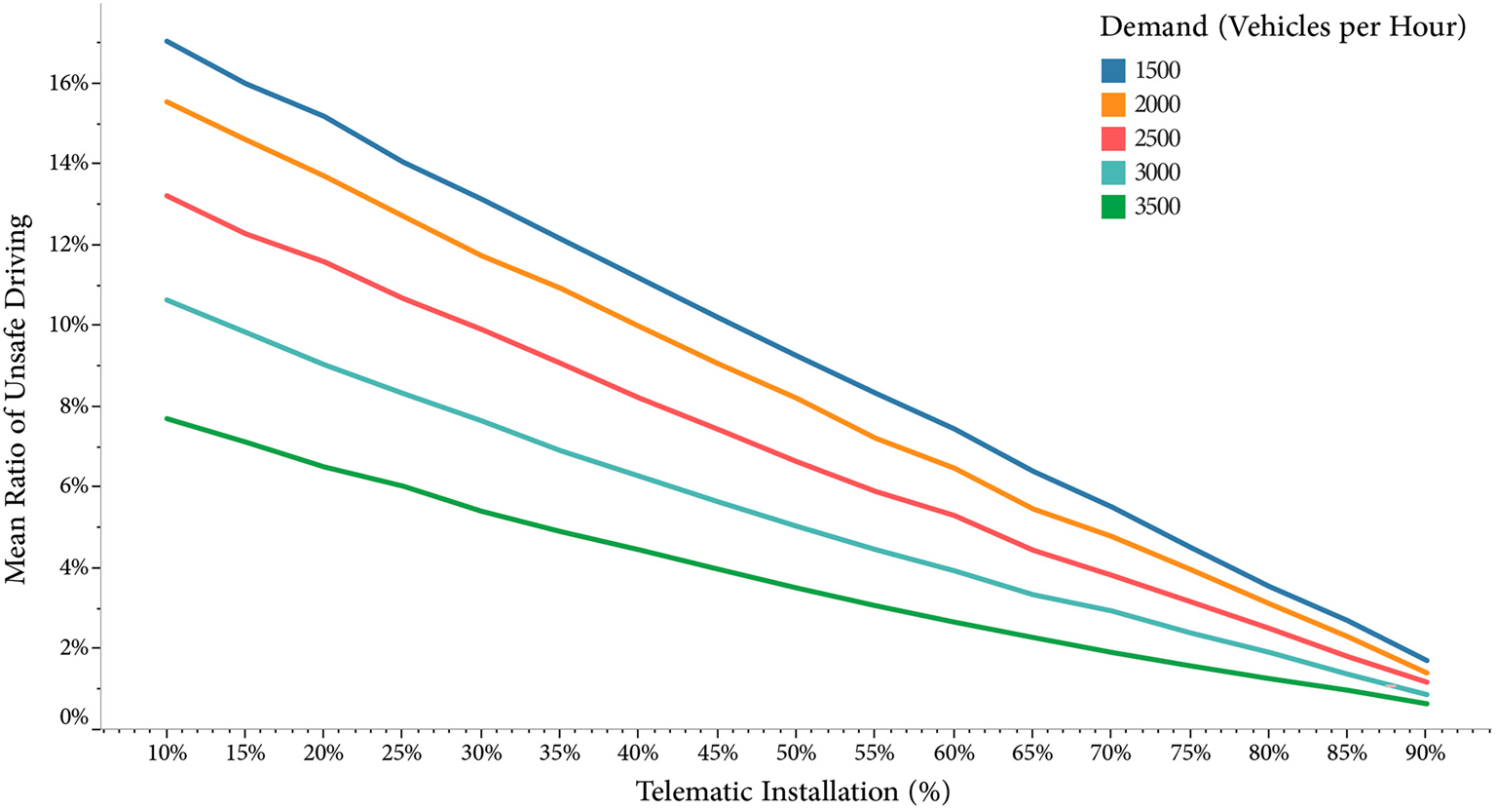
Percent of telematic installation on induvial in agent-based model and ratio of unsafe driving.

In vehicle counts of 500 and 3,500 VPH, the mean RUD was 22.67 (22.59–22.75) and 10.86 (10.81–10.92), respectively (Fig. 3). There was a significant negative association between vehicle count and RUD, with a mean reduction of 3.97% (3.96–3.98) per each 1000 VPH. This inverse association was weaker in VPH of less than 1500 compared to greater VPH, with 1.51% (1.48–1.53) versus 5.79% (5.77–5.81), respectively. Overall vehicle counts levels and the mean RUD of group B and group C had a similar trend and value in fixed SECs (Fig. 4). On the other hand, under average SECs, despite group C had a notably higher mean RUD than group B (58.36% vs. 29.34%), a converging trend was observed at higher vehicle counts. Finally, Fig. 5 shows that the increase of telematics installation on extreme percentages of drivers did not result in the spill-over effect, and RUD followed a relatively linear trend.

### Crash relative risk change

In various scenarios involving the implementation of Speed Enforcement Cameras (SECs), we investigated the relative crash risk associated with different types of SECs, using a single fixed camera as the reference point. As shown in Table 2, the point-to-point camera emerged as the most effective option for reducing crash risk across all scenarios, with reductions ranging from − 3.44% (on a moderate traffic road, in a law-abiding community, with telematic enforcement) to − 11.27% (on a low traffic road, in a law-avoiding community, without telematic enforcement). Additionally, adding one and two fixed SECs to an existing fixed SEC installation on a 6 km stretch of a three-lane highway resulted in reductions in relative crash risk of 0.06–0.29% and − 0.12 to − 0.81%, respectively.

**Table 2:** Crash relative risk change between speed enforcement cameras (SEC) in different implementation scenarios considering community behavior, road traffic, and use of telematics enforcement.

	Community	Traffic	2 Fix SEC vs 1 Fix SEC	3 Fix SEC vs 1 Fix SEC	Point-to-Point SEC vs 1 Fix SEC
			Crash relative risk	Crash relative risk	Crash relative risk
No Telematics	Average	High Traffic	− 0.14%	− 0.28%	− 6.51%
		Low Traffic	− 0.19%	− 0.47%	− 6.81%
	Law-abiding	High Traffic	− 0.06%	− 0.13%	− 3.79%
		Low Traffic	− 0.11%	− 0.27%	− 4.13%
	Law-avoiding	High Traffic	− 0.25%	− 0.58%	− 11.25%
		Low Traffic	− 0.34%	− 0.81%	− 11.27%
	Law-carefree	High Traffic	− 0.14%	− 0.31%	− 6.63%
		Low Traffic	− 0.19%	− 0.47%	− 6.83%
Telematic Enforcement	Average	High Traffic	− 0.11%	− 0.24%	− 5.91%
		Low Traffic	− 0.18%	− 0.42%	− 6.22%
	Law-abiding	High Traffic	− 0.06%	− 0.12%	− 3.44%
		Low Traffic	− 0.11%	− 0.25%	− 3.78%
	Law-avoiding	High Traffic	− 0.23%	− 0.51%	− 10.23%
		Low Traffic	− 0.29%	− 0.73%	− 10.35%
	Law-carefree	High Traffic	− 0.10%	− 0.26%	− 6.01%
		Low Traffic	− 0.18%	− 0.43%	− 6.25%

## Discussion

In this study, we harnessed real-world driving data from proficient drivers in Iran to create an agent-based simulation aimed at evaluating the effectiveness of Speed Enforcement Cameras (SECs). Our research represents a pioneering effort in investigating the influence of SECs on an individual’s entire trip and subsequently translating this impact into crash risk. Our findings reveal that fixed SECs have a minimal impact on the total distance of safe driving, even when the number of cameras is increased. In contrast, average point-to-point SECs exhibit the potential to reduce the Ratio of Unsafe Driving (RUD) by 10% and lower crash relative risk by 4–11% across various scenarios. Notably, point-to-point SECs prove to be approximately 20 times more effective than adding additional fixed SECs on a 6 km three-lane highway. Furthermore, the installation of telematics in high-risk law violators may represent a viable option, with effectiveness falling between that of point-to-point SECs and fixed SECs.

In the subsequent sections, following our discussion of the implications of our results for deployment, we delve into a detailed examination of fixed SEC effectiveness, the behavioral aspects of speed enforcements, and the application of telematic-based tools. Additionally, we emphasize the significance of contextualized enforcement and elucidate our approach for translating simulation results into crash risk, providing a summary of previous efforts in this domain. Our discussion culminates with a mention of the limited strategies of speed enforcement investigated in our work and a consideration of the associated limitations.

Our results suggest that fixed SECs can be effective for hazardous short road sections, while for longer sections, average SECs seem more effective. Highways with low to moderate vehicle demand, as shown in Fig. 4, are better suited for implementing average SECs. Furthermore, Average SECs can be a preventive, acceptable, and cost-effective tool for Road Traffic Incidents^34,35^. Accompanying actions, such as changing the SEC's location, placing warning signs before the SEC, and adopting a proper fine system, can increase driver compliance^35,36^.

Studies evaluating SECs' effect on RTI and speeding face numerous challenges. According to a review of 35 studies, the current literature encounters methodological limitations, moderate heterogeneity, and low quality^15^. Among 24 before-after studies, only ten studies considered controlled analysis for confounders. The consistent finding among them is the strong effect of fixed SECs on speeding and crash prevention within the fixed SEC zone^15,37^, while our results should fixed SEC inefficiency in speed enforcement The reason is that we investigated the RUD throughout a driver’s whole trip on a 6-km highway, not only within the vicinity of the camera zone. Our finding aligns with a few studies investigating the SECs’ effect beyond the camera zone^6,14,28^. Current literature estimates that 80% of drivers exceed the speed limit in the outreach of the camera zone (i.e., 3 km before and after the installed camera)^6,14^. To explain the impact of fixed SEC vanishing outside this zone, we must consider human behavior interaction with enforcement and context.

First, speed enforcement aims at modifying human behavior, which is a complex system. Grounded in protection-motivation theory and discussed by Chen et al., humans will adjust their behavior to avoid threats and punishment^36,38^. This theory explains the motivation of the driver to evade the SEC, a phenomenon known as the distance halo effect or kangaroo driving. Each time an individual evades the SEC, their response efficacy (efficacy of evading a threat) increases, and their desire to evade forthcoming SECs will consequently increase. This way, we can explain the time halo effect and why the effectiveness of SECs decreases over time. Second, studies are grounded in different contexts, leading to heterogeneous findings. RTI is a multifactorial issue related to road safety, vehicle safety, post-accident care, and enforcement^39^. In addition, fines should be disincentive enough to change the driver's behavior and encourage safe driving in a trade-off between fines and potential income^36^.

As we demonstrated in previous investigations, drivers' behavior differs when they encounter enforcement^17,28,29^. We found that Group B (Kangaroo drivers who evade enforcement) and C (careless about enforcement) had similar RUD trends when facing fixed SECs, indicating the inefficacy of this enforcement. Nonetheless, average SECs were preventive enough to change the mean RUD of Group B. Furthermore, a one percent increase in Group B and C proportion in the whole population results in a 0.61% and 0.68% increase in total RUD, respectively. These findings resonate with the community's behavior towards enforcements, as law-abiding communities adhere to regulations as opposed to law-carefree and law-avoiding communities.

Telematics installations can be a future option to further improve road safety in resource-rich contexts, such as countries with high developmental indexes^7,40^. We found that each percent increase in telematic installation can reduce RUD by 0.22%, which is higher than fixed SECs and lower than point-to-point SECs. Compared to SECs, the infrastructure required for implementing telematic systems for enforcement needs more resources, making them an inferior option to average SECs in developing countries^11^. However, the use of telematics systems for preventing RTIs is not limited to speed enforcement; it also extends to various other applications, including traffic management, communication with other systems (e.g., emergency units), and driver assistance systems^7,11^. Additionally, telematics can reduce RTIs through interventions other than incentives, such as providing feedback to drivers at the time of distraction^40–42^. Estimating the total benefit of telematic-based interventions and their cost–benefit balance is challenging, and future studies are warranted to investigate their impact on driving behavior.

Inspired by the motivation to assess the impact of SECs on safe driving, we employed the Power Model to translate mean speed to crash risk across various scenarios. Motivated by the same goal, previous studies also attempted to account for unique road characteristics to determine speed-crash risk, highlighting the gap in translating road safety research into practice^18,43^. Ezra Hauer sincerely addressed this gap in the validity of road research and the lack of evidence-based decision-making^18^. In a pioneering effort, we used aggregated average speed of scenarios and the Power Model to calculate crash relative risk. Our findings strongly suggest that point-to-point SEC is the superior intervention across various scenarios, while most SECs are usually of the fixed type in many developing countries. This ultimately confirms the concern regarding the research-practice gap^18^.

Although the current mainstream evidence supports that average vehicle speeds have a negative association with crash risk^43^, one should consider the threats to the validity of our approach concerning the use of aggregated average speed and the Power Model^5^. It is worth mentioning that our approach aimed to provide tools for decision-makers at a low cost, which may result in limited validity. Our pipeline can serve as an initial result for decision-makers, but they should continue monitoring the interventions using more validated approaches. Future studies are warranted to validate this approach using real-world investigations paralleled with replicating our method.

This study took into account the unique road and socio-economic characteristics by including speed limits, driving behavior, desired free-flow speed, road geometry, and other relevant factors in the modeling process. Given the unique conditions of each roadway and the variations in socio-economic factors, it is essential to conduct data-driven assessments for the implementation of safety countermeasures^18^. Attributes such as road classification, behavior, speed limit, control level, and traffic demand may vary in different conditions and can significantly impact the effectiveness of preventive countermeasures^43^. Therefore, conducting such studies in diverse contexts becomes essential to pinpoint the optimal strategy for each specific situation.

Safety countermeasures are not limited to the strategies studied in this research (i.e., SECs and telematics). Although these solutions were among the more common ones for the studied case, other cost-effective countermeasures exist. For instance, variable speed limits (VSLs) are another applicable safety countermeasure that can be assessed and utilized in high-speed roadways^35,36^. Another example is the Leading Pedestrian Interval (LPI) which is commonly used at signalized intersections to protect vulnerable road users^44^. Therefore, comprehensive analyses should be conducted to identify the most effective strategies and interventions for a given context. There is no one-size-fits-all safety countermeasure that can be suggested for all conditions.

This study has several limitations that should be considered for interpretation. We used the behavior of expert taxi drivers in Iran for our agent-based simulation. Although we explored different scenarios with different percentages of Groups A, B, and C, our simulation may not represent an actual sample of individuals. Additionally, since this study is among the pioneers in using agent-based modeling to assess SEC effectiveness, there is no other study for comparison. Moreover, it is essential to consider that there are additional influential factors contributing to driving behavior heterogeneity in real-world settings that have not been incorporated into this study's model. Nevertheless, our results are roughly aligned with some of the previous investigations. There are multiple important threats regarding the internal, external, and statistical validity of our speed-crash risk translation and Power Model formula, discussed in detail by its creators^5^. It should be noted that the desired methodology for assessing SEC effectiveness is randomized trials with control for confounders, and our approach lacks the most substantial evidence for decision-making. Additionally, the assessment was conducted on a 6-km highway segment with unique characteristics. Other uninterrupted-flow (e.g., freeways) and interrupted-flow (e.g., arterial and collector urban streets) facilities can be tested in future research using a similar methodology to explore the impact of road classification on the obtained results.

## Conclusion

In conclusion, our cost-conscious and replicable approach can serve as an initial step in designing speed enforcement interventions, even in low-income countries. However, it's important to note that the evaluation and revision of interventions may require greater validity and additional resources in the long term. When compared to fixed SECs, the point-to-point average SEC demonstrated approximately 20 times greater effectiveness in reducing RUD and crash relative risk, which may also surpass telematic-based interventions. Policymakers should carefully consider their unique context, taking into account road and socio-economic characteristics when selecting an appropriate strategy. While further validation of our methodology is essential, future studies can enrich our approach by integrating SEC cost components, offering decision makers a comprehensive perspective on intervention cost-effectiveness.

## Data Availability

The datasets used in the current study are available from the corresponding author at a reasonable request. The aggregated simulation results are available at: https://github.com/more-malekpour/telematics-agent-based-simulation.
